# Methyl Caffeate Binds to IQGAP1 and Inhibits the Senescence-Associated Secretory Phenotype in Senescent Cells

**DOI:** 10.3390/ijms27125199

**Published:** 2026-06-09

**Authors:** Shusuke Yasuda, Yosuke Iizumi, Mamiko Sukeno, Toshiyuki Sakai, Mano Horinaka

**Affiliations:** 1 Department of Drug Discovery Medicine, Graduate School of Medical Science, Kyoto Prefectural University of Medicine, Kawaramachi-Hirokoji, Kamigyo-ku, Kyoto 602-8566, Japan; syu-ysd@koto.kpu-m.ac.jp (S.Y.); sukeno@koto.kpu-m.ac.jp (M.S.); tsakai@koto.kpu-m.ac.jp (T.S.); 2 Division of Immunology, Faculty of Medicine, Tohoku Medical and Pharmaceutical University, Fukumuro, Miyagino-ku, Sendai 983-8536, Japan; iizumi.yosuke@tohoku-mpu.ac.jp; 3 Department of Molecular-Targeting Prevention, Graduate School of Medical Science, Kyoto Prefectural University of Medicine, Kawaramachi-Hirokoji, Kamigyo-ku, Kyoto 602-8566, Japan

**Keywords:** cellular senescence, SASP, IQGAP1

## Abstract

The senescence-associated secretory phenotype (SASP) contributes to various age-related pathologies. Methyl caffeate exhibits strong SASP-inhibitory activity; however, its molecular targets and the precise mechanisms underlying its effects remain unclear. Therefore, in this study, we performed affinity chromatography using methyl caffeate-immobilized beads to identify its intracellular binding proteins. The functional roles of the identified target were validated via knockdown experiments, assessment of SASP factor (interleukin [IL]-6 and IL-8) expression at the mRNA and secretion levels, and analysis of nuclear factor-κB and p38 mitogen-activated protein kinase signaling pathways. IQ motif-containing GTPase-activating protein 1 (IQGAP1) was identified as a methyl caffeate-binding partner. *IQGAP1* knockdown significantly reduced IL-6 and IL-8 expression levels, mimicking the effects of methyl caffeate treatment. Furthermore, IQGAP1 depletion suppressed nuclear factor-κB activation and p38 phosphorylation. Overall, this study identified IQGAP1 as a critical scaffold protein essential for SASP induction and a target of methyl caffeate. Our findings provide key insights into SASP regulation, facilitating the development of SASP-modulating therapeutics targeting specific IQGAP1 domains.

## 1. Introduction

Cellular senescence is a phenomenon characterized by irreversible cell cycle arrest induced in response to various stressors, such as DNA damage, and it serves as a critical defense mechanism against oncogenesis [1]. Senescent cells exacerbate the surrounding tissue microenvironment via secretion of the senescence-associated secretory phenotype (SASP) [1,2,3]. This chronic exposure to SASP factors promotes pathological conditions such as tissue fibrosis and chronic inflammation [4,5]. Furthermore, it drives cancer progression by inducing epithelial–mesenchymal transition and invasion in breast cancer cells [6], as well as obesity-associated hepatocellular carcinoma development in mice [7]. Unlike senolytics, which selectively remove senescent cells, senomorphics, which suppress only the harmful secretory function of senescent cells, are expected to suppress only pathological aspects while maintaining tissue repair capacity [8,9,10,11].

The transcription factor nuclear factor (NF)-κB plays a central role in SASP induction [12,13]. In senescent cells, stress response signals such as the p38 mitogen-activated protein kinase (MAPK) pathway are activated. Subsequently, downstream NF-κB nuclear translocation and transcriptional activity are promoted, leading to the comprehensive expression of inflammatory cytokines and chemokines [11,14,15]. Therefore, suppressing these upstream SASP inducers via senomorphics is an effective strategy to prevent the development and progression of malignant tumors induced by senescent cells.

While several drugs and natural compounds, such as rapamycin, metformin, and methyl caffeate, have been identified as senomorphics inhibiting SASP [16,17,18,19,20,21], the precise mechanism is unknown. Especially, the intracellular target proteins to which these SASP inhibitors bind are largely unknown. Identification of SASP inhibitor targets may facilitate the development of novel molecular-targeted agents that specifically suppress SASP. Furthermore, it may be possible to predict the effects of target molecules based on their information. In order to clarify the molecular mechanisms of senomorphics, we focused on methyl caffeate, a compound previously reported to exhibit potent SASP-inhibitory activity [21]. Using methyl caffeate-immobilized beads, we aimed to identify its intracellular binding proteins that function as biological effectors, thereby contributing to a clearer understanding of potential drug targets for SASP suppression.

## 2. Results

### 2.1. Methyl Caffeate Inhibits SASP by Decreasing NF-κB Activity and p38 Phosphorylation

SASP is involved in carcinogenesis and cancer promotion [22,23,24]; its inhibition possibly prevents cancer. To elucidate the cancer prevention mechanisms of components with SASP-inhibitory activity, we searched for SASP inhibitor-binding proteins in cells.

**Figure 1:**
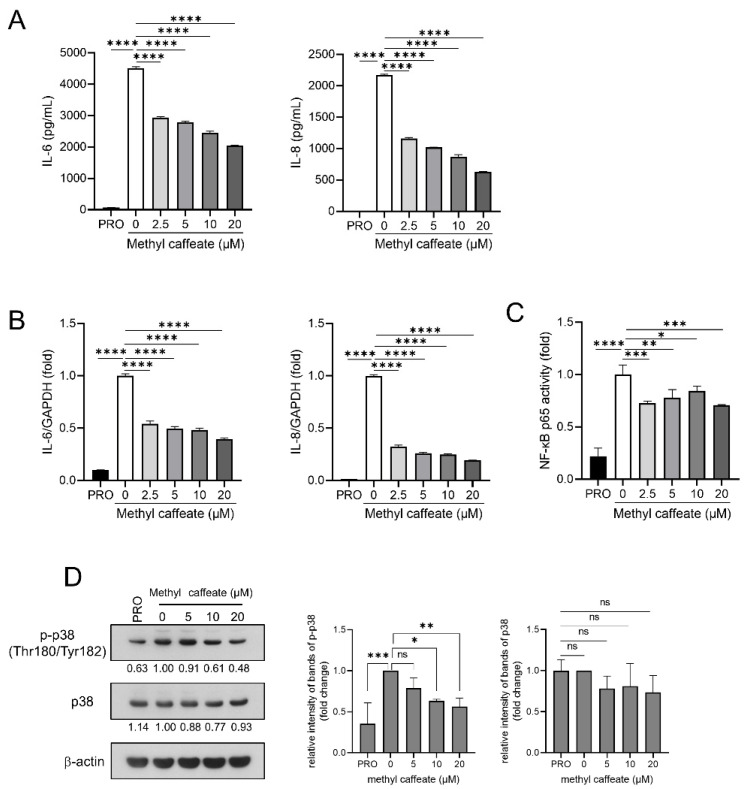
Methyl caffeate inhibits SASP and reduces NF-κB activity and p38 phosphorylation. BJ fibroblasts were treated with the indicated concentrations of methyl caffeate for 24 h, followed by bleomycin treatment for 24 h to induce senescence. Then, the cells were washed, replenished with fresh medium, and cultured for 144 h. BJ fibroblasts not treated with bleomycin were used as proliferative cells (PRO). (**A**) Secretion levels of IL-6 and IL-8 in the culture supernatants of bleomycin-induced senescent cells. (**B**) mRNA expression levels of *IL-6* and *IL-8* in bleomycin-induced senescent cells. (**C**) NF-κB p65 activity in bleomycin-induced senescent cells. (**D**) Immunoblotting analysis of phospho-p38 and p38 levels in bleomycin-induced senescent cells. β-actin was used as an internal control. The band intensities were quantified using ImageJ software and normalized using that of β-actin. Data are represented as the mean ± standard deviation (SD; *n* = 3). *p* values were calculated using one-way analysis of variance (ANOVA): * *p* < 0.05, ** *p* < 0.01, *** *p* < 0.001, and **** *p* < 0.0001.

We focused on methyl caffeate, previously reported to exhibit SASP-inhibitory activity [8], and confirmed its activity in senescent cells. Senescence was induced in BJ human fibroblasts via 50 μg/mL bleomycin treatment for 24 h (Supplementary Figure S1). The culture supernatants were collected 144 h after senescence induction, and the levels of the SASP factors interleukin (IL)-6 and IL-8 were measured via enzyme-linked immunosorbent assay (ELISA). As shown in Figure 1A, senescence induction increased the amount of SASP factors secreted by cells into the culture medium. However, pretreatment with methyl caffeate dose-dependently reduced this secretion (Figure 1A). Similarly, senescence increased *IL-6* and *IL-8* mRNA expression levels; however, pretreatment with methyl caffeate reduced their levels (Figure 1B). As NF-κB regulates SASP, we examined the effect of methyl caffeate on NF-κB. Notably, pretreatment with methyl caffeate reduced the senescence-induced activation of the NF-κB p65 subunit (Figure 1C). We further investigated whether methyl caffeate inhibits the pathways contributing to SASP induction. Methyl caffeate reduced phosphorylated p38 protein levels in senescent cells (Figure 1D).

### 2.2. Methyl Caffeate Binds to IQ Motif-Containing GTPase-Activating Protein 1 (IQGAP1)

**Figure 2:**
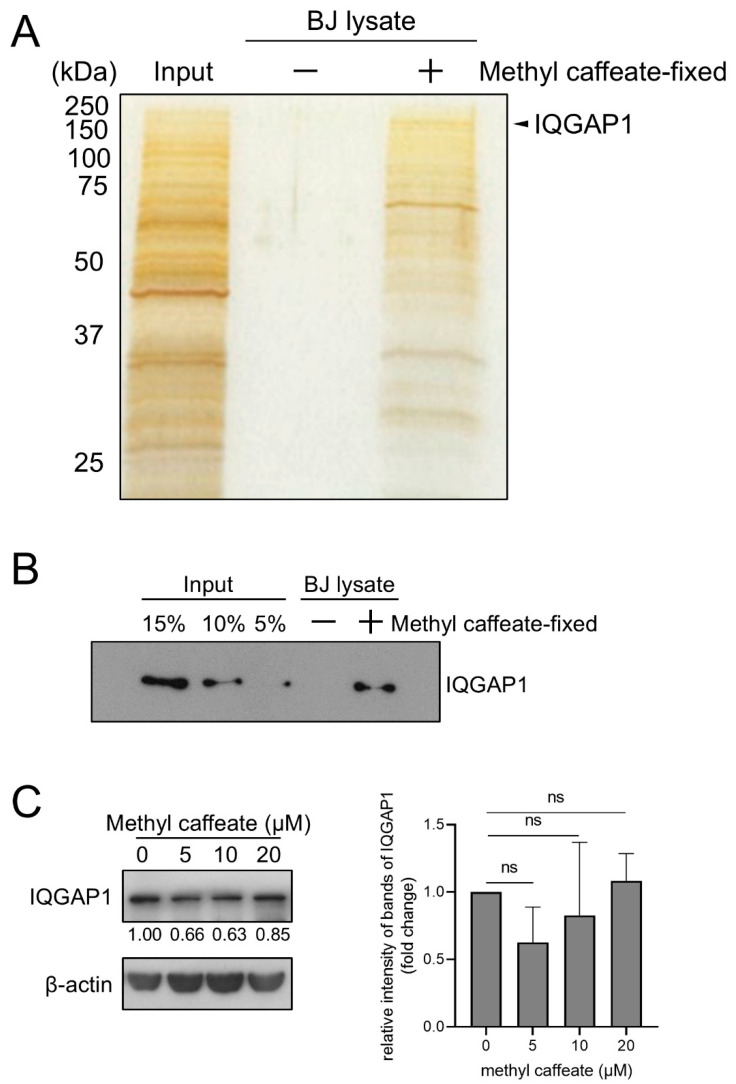
Methyl caffeate binds to IQ motif-containing GTPase-activating protein 1 (IQGAP1). (**A**) Methyl caffeate-binding proteins were purified from whole-cell extracts of BJ fibroblasts using methyl caffeate-immobilized (+) or empty (−) beads and detected via silver staining. Mass spectrometry identified IQGAP1 as a methyl caffeate-binding protein. Input: Whole-cell extracts of BJ fibroblasts. (**B**) Confirmation of mass spectrometry results via immunoblotting using anti-IQGAP1 antibody. Input: Whole-cell extracts of BJ fibroblasts. (**C**) Immunoblotting analysis of IQGAP1 levels in bleomycin-induced senescent cells treated with methyl caffeate. β-actin was used as an internal control. The band intensities were quantified using ImageJ software and normalized using that of β-actin. Data are represented as the mean ± standard deviation (SD; *n* = 3).

To determine the mechanisms by which methyl caffeate inhibits SASP, we identified the proteins binding to it using FG beads. Methyl caffeate-immobilized beads were incubated at 4 °C with whole-cell extracts of BJ fibroblasts. Silver staining of purified methyl caffeate-binding proteins revealed several bands, one of which was identified as IQGAP1 via matrix-assisted laser desorption/ionization time-of-flight mass spectrometry analysis (Figure 2A, Supplementary Figure S2). Subsequently, we confirmed the binding of IQGAP1 via Western blotting using a specific antibody (Figure 2B). The results indicated that methyl caffeate bound to IQGAP1. Notably, methyl caffeate did not reduce IQGAP1 expression in bleomycin-induced senescent cells (Figure 2C).

### 2.3. IQGAP1 Knockdown Inhibits SASP in Senescent Cells

**Figure 3:**
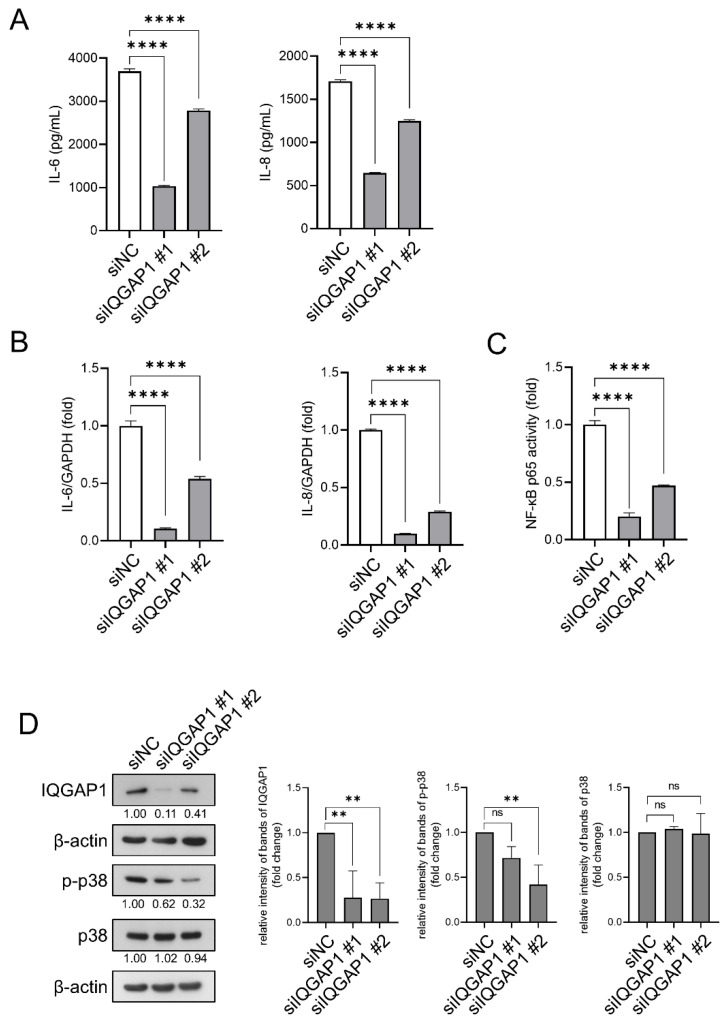
*IQGAP1* knockdown inhibits SASP and reduces NF-κB activity and p38 phosphorylation. BJ fibroblasts were transfected with control small interfering RNA (siRNA; siNC) or two independent *IQGAP1* siRNAs for 24 h, followed by bleomycin treatment for 24 h to induce senescence. The cells were washed and cultured in fresh medium for an additional 144 h. (**A**) Secretion levels of IL-6 and IL-8 in the culture supernatants of bleomycin-induced senescent cells. (**B**) mRNA expression levels of *IL-6* and *IL-8* in bleomycin-induced senescent cells. (**C**) NF-κB p65 activity in bleomycin-induced senescent cells. (**D**) Immunoblotting analysis of phospho-p38 and p38 levels in bleomycin-induced senescent cells. β-actin was used as an internal control. The band intensities were quantified using ImageJ software and normalized using that of β-actin. Data are represented as the mean ± SD (*n* = 3). *p* values were calculated using one-way ANOVA: ** *p* < 0.01, **** *p* < 0.0001.

To determine whether IQGAP1 is involved in SASP in senescent cells, we investigated whether *IQGAP1* knockdown inhibits SASP. As shown in Figure 3A, *IQGAP1* knockdown reduced IL-6 and IL-8 levels in the culture supernatants of bleomycin-induced senescent cells. *IQGAP1* knockdown also reduced *IL-6* and *IL-8* mRNA levels in senescent cells (Figure 3B). Additionally, *IQGAP1* knockdown reduced the activity of the NF-κB p65 subunit induced by bleomycin (Figure 3C). We also measured phosphorylated p38 levels via Western blotting. Similar to methyl caffeate treatment, *IQGAP1* knockdown reduced phosphorylated p38 levels, while total p38 expression remained unaltered in senescent cells. Importantly, both siRNA treatments resulted in a similar decreasing trend in phosphorylated p38 levels, one of which was statistically significant (Figure 3D).

## 3. Discussion

Identifying the targets of SASP inhibitors is necessary to elucidate the molecular mechanisms underlying SASP regulation and facilitate the development of specific SASP inhibitors. In this study, we used beads immobilized with methyl caffeate, which exhibits potent SASP-inhibitory activity [21], and identified the intracellular binding protein IQGAP1 (Figure 2). *IQGAP1* knockdown reduced the secretion and mRNA expression levels of *IL-6* and *IL-8*, decreased NF-κB activity, and showed a decreasing trend in p38 phosphorylation (Figure 3). These phenotypic and molecular changes induced by *IQGAP1* knockdown were consistent with the effects observed with methyl caffeate treatment (Figure 1).

Previous literature indicates that IQGAP1 has multiple binding domains and regulates the functions of more than 90 proteins [25,26]. It regulates signaling efficiency by bringing components of the MAPK signaling pathway into proximity [27,28,29]. Furthermore, it has been reported that *IQGAP1* knockdown inhibits NF-κB translocation to the nucleus and NF-κB activity [30,31]. Based on these reported functions and our current findings, we hypothesize that IQGAP1 functions as a critical scaffold protein necessary for the activation of p38 and NF-κB, thereby inducing the transcription of SASP factor in bleomycin-induced senescent cells. Considering its multidomain structure, it is conceivable that IQGAP1 exerts either pro- or anti-SASP effects depending on the specific binding site targeted. Therefore, we propose that methyl caffeate suppresses SASP potentially by binding to a specific domain of IQGAP1, thereby disrupting the downstream proinflammatory signaling. Elucidating the precise binding site of methyl caffeate on IQGAP1 will be required to clarify the molecular mechanisms underlying SASP and provide crucial insights for the development of novel SASP-modulating therapeutics.

As shown in Figure 1, pretreatment with methyl caffeate reduced the mRNA levels of the SASP factors *IL-6* and *IL-8* and decreased NF-κB activation and p38 phosphorylation. In a previous study, treating cells with methyl caffeate for 24 h on day 5 following 24 h exposure to bleomycin suppressed IL-6 secretion [21]. In contrast, in this study, pretreatment with methyl caffeate for 24 h before senescence induction resulted in SASP inhibition lasting up to 144 h (Figure 1). This result raises the possibility that methyl caffeate is not an enzyme inhibitor but rather potentially disrupts or destabilizes the formation of the IQGAP1-centered complex in the early stages of senescence.

Compared to senescent cell-scavenging agents, naturally derived senomorphics such as methyl caffeate are expected to serve as a safer treatment option that targets only senescence-related adverse events, including SASP. In conclusion, we identified IQGAP1 as a binding protein for the specific SASP inhibitor methyl caffeate. Our findings provide insights into the molecular mechanisms underlying SASP regulation and facilitate the development of next-generation SASP-regulating drugs with enhanced affinity and specificity. A limitation of this study is that, in order to fully characterize binding affinity and kinetics, biophysical and biochemical validation of the interaction between methyl caffeate and IQGAP1 is required through techniques such as surface plasmon resonance, isothermal titration calorimetry, or mutant analysis. Furthermore, while this study provides solid evidence using a senescent BJ cell line model, in order to confirm the generalizability of our findings, it is necessary to extend these findings to a broader range of cellular models (such as different cell lines and primary cultured cells).

## 4. Materials and Methods

### 4.1. Reagents and Antibodies

Bleomycin (#13877) was purchased from Cayman Chemical (Ann Arbor, MI, USA) and dissolved in Milli-Q water (Millipore, Bedford, MA, USA). Methyl caffeate (#M2519) was purchased from Tokyo Chemical Industry (Tokyo, Japan) and dissolved in dimethyl sulfoxide (#13407-45, Nacalai Tesque, Kyoto, Japan). Antibodies against IQGAP1 (#2293), p38 (#9212), and phospho-p38 (Thr180/Tyr182) (#9215) were purchased from Cell Signaling Technology (Danvers, MA, USA). Antibody against β-actin (#A5441) was purchased from Sigma-Aldrich (Saint Louis, MO, USA). Horseradish peroxidase (HRP)-linked donkey anti-rabbit IgG (#NA934) and HRP-linked sheep anti-mouse IgG (#NA931) were purchased from Cytiva (Tokyo, Japan).

### 4.2. Cell Culture

BJ human fibroblasts were obtained from the American Type Culture Collection (Manassas, VA, USA). The cells were maintained in E-MEM (#055-08975; FUJIFILM Wako Pure Chemical Corporation, Osaka, Japan) supplemented with 10% (*v*/*v*) fetal bovine serum (Cat #10437028; Lot #2075863; Thermo Fisher Scientific, Waltham, MA, USA), 50 U/mL of penicillin G (Meiji Seika Pharma. Co., Ltd., Tokyo, Japan), and 100 μg/mL of streptomycin (#06339-52, Nacalai Tesque). The cells were incubated at 37 °C in a humidified atmosphere containing 5% CO_2_.

### 4.3. Proliferative Cells (PRO)

BJ fibroblasts were seeded in 6-well plates or 100 mm dishes and incubated overnight at 37 °C. After replacing the culture medium with fresh medium, the cells were incubated at 37 °C for 24 h. Finally, the cells were collected and used as proliferative cells.

### 4.4. Cellular Senescence Induction

BJ fibroblasts were seeded in 6-well plates or 100 mm dishes and incubated overnight at 37 °C. The cells were treated with dimethyl sulfoxide or methyl caffeate for 24 h. The culture medium was replaced with fresh medium, and the cells were treated with 50 μg/mL bleomycin for 24 h to promote damage-induced senescence. After washing with phosphate-buffered saline and replacing the culture medium with fresh medium, the cells were incubated at 37 °C for 144 h.

### 4.5. ELISA

The culture supernatants were collected 144 h after senescence induction and stored at −80 °C. IL-6 and IL-8 levels in the supernatants were measured using the LBIS Human IL-6 (#294-87401) and LBIS Human IL-8 (CXCL8; #296-87601) ELISA kits (FUJIFILM Wako Pure Chemical Corporation), respectively, according to the manufacturer’s instructions.

### 4.6. Real-Time Reverse Transcription Quantitative-Polymerase Chain Reaction (RT-qPCR)

Total RNA was isolated from the cells using Sepasol-RNA I Super G (#09379-84; Nacalai Tesque), according to the manufacturer’s instructions. Then, the total RNA was reverse-transcribed to complementary DNA (cDNA) using the ReverTra Ace qPCR RT Master Mix (#FSQ-201; TOYOBO, Osaka, Japan). An equivalent volume of cDNA solution was used for quantitative RT-PCR. cDNA was amplified using the QuantStudio 3 Real-Time PCR System (Thermo Fisher Scientific) with TaqMan Probes for *IL-6* (Hs00985639), *CXCL8* (Hs00174103), and glyceraldehyde-3-phosphate dehydrogenase (GAPDH) (Hs02758991). The expression of each mRNA was normalized to that of GAPDH mRNA in the same sample. The experiment was conducted based on reference [32].

### 4.7. NF-κB p65 Activity Assay

Using the Nuclear Extract Kit (#40010; Active Motif, Carlsbad, CA, USA), the cells were lysed, and the nuclear extracts were collected. Subsequently, the nuclear extracts were quantified using the TransAM NF-κB p65 Activity Assay (#40096; Active Motif), according to the manufacturer’s instructions.

### 4.8. Western Blotting

The cells were lysed in a buffer containing 50 mM Tris-HCl (pH 7.5), 1% sodium dodecyl sulfate, a protease inhibitor cocktail (#25955-24; Nacalai Tesque), and a phosphatase inhibitor cocktail (#07575-51; Nacalai Tesque). The lysate was sonicated and centrifuged at 14,000× *g* for 20 min at 4 °C, and the supernatant was collected. Equal amounts of lysates were analyzed via SDS-PAGE and transferred to polyvinylidene difluoride membranes (#IPVH00010; Millipore). The blots were blocked with 3% bovine serum albumin (Nacalai Tesque) for 1 h at room temperature, followed by incubation with the appropriate primary antibody in blocking buffer overnight at 4 °C. The blots were washed with Tris-buffered saline (20 mM Tris-HCl, pH 7.5, 150 mM NaCl) containing 0.05% Tween-20 and incubated with the appropriate HRP-conjugated secondary antibody at room temperature for 1 h, and signals were detected using Chemi-Lumi-One chemiluminescent kit (#07880-70; Nacalai Tesque) or Immobilon Western Chemiluminescent HRP Substrate (#WBKLS0500; Millipore). The band intensities of the Western blot were measured using ImageJ 1.50i (National Institutes of Health, Bethesda, MD, USA). The experiment was conducted based on reference [33].

### 4.9. Immobilization of Methyl Caffeate onto FG Beads

FG beads with epoxy linkers (#TAS8848N1110) were purchased from Tamagawa Seiki (Nagano, Japan). Methyl caffeate was immobilized onto the beads with potassium carbonate, as previously described [34]. Briefly, the beads were mixed with methyl caffeate in N,N-dimethylformamide (DMF) containing potassium carbonate at 37 °C for 24 h. After washing twice with DMF, they were washed twice with deionized water and stored at 4 °C until use.

### 4.10. Purification and Identification of Methyl Caffeate-Binding Proteins

Whole-cell extracts of 0.1 mg of BJ fibroblasts were prepared with NP-40 lysis buffer (50 mM Tris-HCl [pH 8.0], 150 mM NaCl, 1% NP-40, 1 mM dithiothreitol, and 0.5 mM phenylmethylsulfonyl fluoride), as previously described [35]. The resulting lysates were incubated with 0.1 mg of methyl caffeate-fixed beads or empty beads for 4 h at 4 °C. Following incubation, the beads were washed three times with 400 μL of binding buffer (50 mM Tris-HCl [pH 8.0], 150 mM sodium chloride, 0.1% NP-40) to remove non-specific interactors. The bound proteins were eluted using Laemmli SDS sample buffer (#J60015; Thermo Fisher Scientific), resolved via 12% SDS-PAGE, and visualized by silver staining. Each gel slice containing a methyl caffeate-binding protein was subjected to in-gel digestion with Sequencing Grade Modified Trypsin (Promega, Madison, WI, USA). The peptide fragments were identified using an Autoflex II (Bruker Daltonics, Billerica, MA, USA), as previously described [35].

### 4.11. Small Interfering RNA (siRNA) Transfection

BJ fibroblasts were seeded in 6-well plates and incubated overnight at 37 °C. siRNAs against *IQGAP1* (IQGAP1-HS113014 containing the following siRNA target sequences: 5′-UUUAGCUGCAGGAAUCUGUAGGGCC-3′ and 5′-GGCCCUACAGAUUCCUGCAGCUAAA-3′: #1 and Silencer Select Pre-Designed siRNA s16838 targeting the sequence: 5′-GUACUUCCGGGACCAUAUATT-3′ and 5′-UAUAUGGUCCCGGAAGUACUG-3′: #2) and Negative Control Medium GC Duplex #2 (Invitrogen, Carlsbad, CA, USA) were transfected into the cells using Lipofectamine RNAiMAX (#13778150; Invitrogen), according to the manufacturer’s instructions. Twenty-four hours after transfection, the culture medium was replaced with fresh medium, and the cells were treated with 50 μg/mL bleomycin for 24 h to promote damage-induced senescence. After washing with phosphate-buffered saline and changing the culture medium to fresh medium, the cells were incubated at 37 °C for 144 h.

### 4.12. Statistical Analyses

Experimental values are presented as the mean ± standard deviation of three determinations. Data were analyzed to determine statistical significance using one-way analysis of variance, followed by Dunnett’s or Sidak’s test, and two-way analysis of variance, followed by Tukey’s multiple comparisons test. Statistical analyses were performed using GraphPad Prism version 8.4.3 (GraphPad Software, Boston, MA, USA).

## Data Availability

The original contributions presented in this study are included in the article/Supplementary Materials. Further inquiries can be directed to the corresponding author.

## Supplementary Materials

The following supporting information can be downloaded at https://www.mdpi.com/article/10.3390/ijms27125199/s1.

## Abbreviations

The following abbreviations are used in this manuscript:

SASP	Senescence-associated secretory phenotype
IL	Interleukin
IQGAP1	IQ motif-containing GTPase-activating protein 1
NF-κB	nuclear factor-κB
MAPK	mitogen-activated protein kinase
HRP	Horseradish peroxidase
ELISA	Enzyme-linked immunosorbent assay
DMF	N,N-dimethylformamide
