# Supplementary material for: Methyl Caffeate Binds to IQGAP1 and Inhibits the Senescence-Associated Secretory Phenotype in Senescent Cells
Source: Int J Mol Sci. 2026 Jun 9;27(12):5199. doi: 10.3390/ijms27125199 (PMC13299695; doi:10.3390/ijms27125199)
Supplement: Supplementary file 1 [file ijms-27-05199-s001.zip › ijms-4316271-supplementary.pdf]

Supplementary Materials:

Supplementary Methods

Senescence-associated β-galactosidase (SA-β-gal) **assay**

SA-β-gal activity was assayed using the Senescence Detection Kit (BioVision, Milpitas, CA, USA) according to manufacturer's instruction.

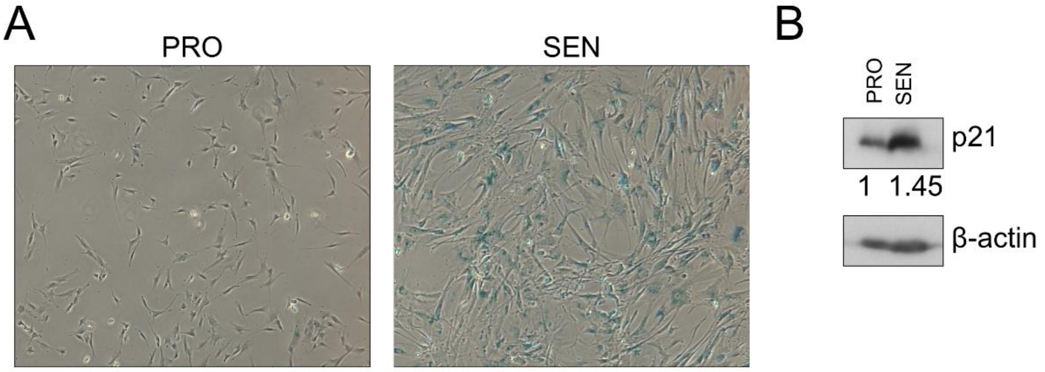

Supplementary Figure S1. Bleomycin induces cellular senescence in BJ cells.

BJ fibroblasts were seeded and incubate for 24 h, followed by bleomycin treatment for 24 h to induce senescence. Then, the cells were washed, replaced to fresh medium, and cultured for 144 h (senescent cells, SEN). BJ fibroblasts not treated with bleomycin were used as proliferative cells (PRO). (A) Senescence associated β-galactosidase (SA-β-gal) activity of senescent cells was assayed using Senescence Detection kit. (B) The expression of the senescent cell marker p21 was identified by western blotting. β-actin is a loading control. The band intensities were quantified using ImageJ software and normalized using that of β-actin.

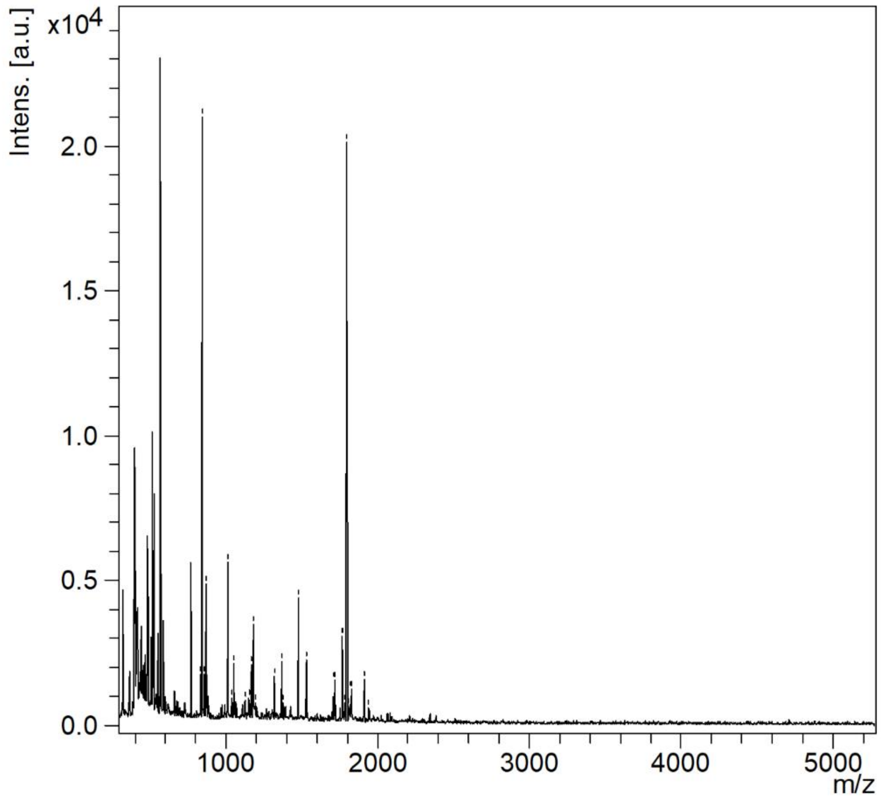

Supplementary Figure S2, The mass spectrum of IQGAP1.
